# The Association Between Physical Exercise and Local Cultural Identity: Indirect Associations Through Three Dimensions of Social Support

**DOI:** 10.3390/bs16091696

**Published:** 2026-09-20

**Authors:** Boqian Sun, Ye Zhang, Yifei Chen, Xiaoming Wang

**Affiliations:** 1 School of Physical Education, Liaoning Normal University, Dalian 116029, China; sunbq@lnnu.edu.cn (B.S.); chen_jl@lnnu.edu.cn (Y.C.); 2 Department of Physical Education, Guilin University of Technology at Nanning, Nanning 530001, China

**Keywords:** physical exercise, local cultural identity, social support, indirect associations

## Abstract

Using the Physical Activity Rating Scale (PARS-3), the College Students’ Local Cultural Identity Scale, and the Social Support Rating Scale (SSRS), this study surveyed 412 college students to examine the associations among physical exercise, three dimensions of social support (objective support, subjective support, and support utilization), and local cultural identity, with particular attention to the indirect associations through the three dimensions of social support. The results showed that physical exercise was positively associated with local cultural identity. Among the three dimensions of social support, only objective support showed a significant positive association with local cultural identity, whereas subjective support and support utilization did not show significant independent associations with local cultural identity. The total indirect association through the three dimensions of social support was statistically significant. Specifically, the indirect association between physical exercise and local cultural identity through objective support was significant, whereas the indirect associations through subjective support and support utilization were not significant. These findings suggest that objective support may represent one pathway linking physical exercise with local cultural identity among college students. The findings provide implications for understanding the relationship among physical exercise, social support, and local cultural identity in Chinese college students.

## 1. Introduction

Since the 18th National Congress of the Communist Party of China, General Secretary Xi Jinping has positioned the “consolidation of the consciousness of the Chinese nation as the main thread of the Party’s ethnic work in the new era” from the strategic height of realizing the great rejuvenation of the Chinese nation. The core of this initiative is to strengthen the ideological foundation of cultural identity. General Secretary Xi Jinping emphasized, “Cultural identity is the deepest form of recognition; it is the root of ethnic unity and the soul of ethnic harmony” (Chen, 2021). Faced with the surging tide of globalization and the impact of multiculturalism, the common challenge and consideration for all ethnic groups in China is how to maintain and enhance their identification with Chinese culture (Han, 2010). This will truly unleash the centripetal force, cohesive power, and appeal of traditional Chinese culture, elevate China’s cultural soft power, and contribute to the great rejuvenation of the Chinese nation.

Conceptually, “identity” refers to self-recognition—the inquiry and contemplation of questions such as “Who am I, or who are we? Where do we come from? Where are we headed?” (Zhou, 2008). As a means of defining identity, cultural identity reflects an individual’s subjective self-awareness in relation to their cultural group (Patricia, 2010). It represents an individual’s self-concept and self-perception within a specific cultural community (Schwartz et al., 2006). Formed through the interaction between the individual and the cultural context (Padilla & Perez, 2003), it embodies an individual’s sense of belonging and psychological commitment to a particular nation or ethnic group (Berry, 1999). It reflects the degree to which an individual identifies with a particular culture, representing the extent to which their attitudes, perceptions, and behaviors align with those of the majority within that cultural group (Zheng & Wang, 2005). Thus, cultural identity expresses collective recognition of a culture and embodies shared group identity (C. C. Sun et al., 2018). In the context of the present study, “local cultural identity” refers to college students’ identification with Chinese culture, consistent with the construct operationalized by S. J. Liu’s (2019) College Student Local Cultural Identity Scale. This construct comprises three dimensions: cultural affect, reflecting emotional attachment to Chinese culture; cultural behavior, reflecting behavioral engagement with Chinese culture; and cultural confidence, reflecting confidence in the value and vitality of Chinese culture. These three dimensions therefore provide the operational framework through which local cultural identity is examined in the present study. Evidently, an individual’s identification with their native culture not only concerns the construction of self-identity and belonging, but also serves as the foundation for the healthy development of a nation and its people. However, the issue of local cultural identity among China’s university students is currently serious. In 2016, the China Traditional Culture Research Center conducted a survey on the recognition of traditional culture among college students in China. The results revealed that only 26% of respondents believed they possessed sufficient understanding of traditional culture, while a mere 10% held an optimistic outlook on the future development of Chinese traditional culture. These findings indicate that the vast majority of university students exhibit low levels of cultural identity with traditional culture and lack accurate understanding of it, leading to pessimistic attitudes toward the future development trend of traditional culture (S. J. Liu, 2019). Therefore, fostering cultural identity among China’s university students has become an urgent priority.

In recent years, leveraging sports to foster individual cultural identity has garnered widespread attention across sectors. For instance, the European Union has established strategies through white papers to utilize sports in developing cultural identity among immigrant and minority groups (Commission of the European Communities, 2007). The attention given to the relationship between sport participation and individual cultural identity may partly reflect the social and interpersonal experiences associated with sport, including moral and social values (Elbe et al., 2018; Rudd, 2005) and interpersonal skills (C. Sun & Zhang, 2020). On the other hand, sports provide participants with more opportunities to practice cooperative and fair competitive behaviors with others, thereby significantly advancing the process of social integration (Donnelly & Donnelly, 2002).

Unlike foreign studies focusing on immigrants’ cultural identity, Chinese research prioritizes leveraging sports to foster domestic public recognition of traditional Chinese culture (J. Liu, 2021; C. C. Sun et al., 2018). These studies either conduct macro-level analyses of the relationship between sports and cultural identity from political science or sociology perspectives or explore the role of sports activities in developing group cultural identity through anthropological and ethnological lenses, reflecting multifaceted, multidimensional, and multilevel approaches to this issue. Taken together, previous research provides evidence that participation in physical exercise or sport is associated with cultural identity and that physical exercise is also related to social support. These findings provide a basis for considering social support as a potential link between physical exercise and local cultural identity. However, several issues remain uncertain. First, little empirical research has directly examined the association between physical exercise and local cultural identity among Chinese university students. Second, although previous studies have separately reported associations between physical exercise and social support, between social support and cultural identity, and between physical exercise or sport participation and cultural identity, these pairwise relationships have rarely been integrated within a single analytical framework. To our knowledge, no previous study has simultaneously examined physical exercise, the three dimensions of social support—objective support, subjective support, and support utilization—and local cultural identity within a single model. Consequently, it remains unclear whether these dimensions of social support represent distinct indirect pathways in the association between physical exercise and local cultural identity.

To address these gaps, the present study simultaneously examined objective support, subjective support, and support utilization as three parallel mediators in the association between physical exercise and local cultural identity among Chinese university students. Rather than treating social support only as a global construct, this multiple-mediator approach allowed us to estimate the specific indirect association through each dimension while accounting for the other dimensions in the same model. In this way, the study extends previous research by integrating physical exercise, multidimensional social support, and local cultural identity within a single analytical framework and by examining whether the three dimensions of social support show differential indirect associations. Accordingly, the present study aimed to examine the association between physical exercise and local cultural identity among Chinese university students and to investigate the specific indirect associations through objective support, subjective support, and support utilization within a parallel multiple-mediator model.

### 1.1. Physical Exercise and Cultural Identity

Based on research methodology characteristics, studies in this field primarily fall into two categories. The first category is cross-sectional studies. Multiple cross-sectional studies have confirmed a significant positive correlation between sports activities and cultural identity. These studies found that participating in sports increases communication and interaction between immigrant groups and members of the mainstream group, helping to establish closer relationships between the two.

This enables individuals to gain more social support, allowing them to better integrate into mainstream society (Ito et al., 2011; Lee, 2005; Rosenberg et al., 2003). The second category is intervention studies. To better leverage physical activity’s positive role in developing individual cultural identity, researchers used sports as a means to intervene in individuals’ cultural adaptation processes. These studies further reported associations between physical activity or sport participation and cultural identity. For example, Guerin et al. provided Somali female refugees with diverse sports activities—including community center exercise classes, fitness club sessions, and walking groups—to help them better adapt to New Zealand’s sociocultural life. After participating in different forms of physical activities, participants reported that these activities provided more opportunities to interact with women of other ethnicities within the community, while also fostering interpersonal relationships and cohesion within their communities (Guerin et al., 2003).

Although these studies primarily concern migrants or minority groups adapting to a host or mainstream culture, they are relevant to the present study at a more general theoretical level. Their relevance lies not in assuming that cultural integration and identification with one’s existing culture are equivalent outcomes, but in suggesting that participation in physical exercise or sport may be associated with social experiences through which individuals relate to cultural groups and communities. Cultural identity, whether examined in the context of adaptation to a new cultural group or identification with one’s existing cultural community, involves individuals’ perceived connection, belonging, and psychological attachment to a cultural group. Accordingly, previous findings from migrant and minority populations provide a conceptual basis for examining whether physical exercise is also associated with identification with one’s existing cultural community. However, they cannot be directly generalized to local cultural identity among Chinese university students, and this relationship therefore requires direct empirical examination in the present population. More recent research has also examined the relationship between sport participation and identity-related outcomes among Chinese university students. For example, Ji et al. (2024) reported a positive association between sport participation and broader social identity in this population. Although social identity is conceptually distinct from the local cultural identity examined in the present study, this finding provides recent evidence that sport participation may be associated with identity-related outcomes among Chinese university students. Despite evidence linking physical exercise or sport participation with cultural identity in other populations and contexts, empirical evidence specifically concerning the association between physical exercise and local cultural identity among Chinese university students remains limited. Based on the theoretical and empirical evidence reviewed above, the following hypothesis was proposed: H1. Physical exercise is positively associated with college students’ local cultural identity.

### 1.2. Physical Exercise, Social Support, and Cultural Identity

Social support refers to the verbal, behavioral, resource, and emotional encouragement and support an individual experiences within their social network or field (Malecki & Demaray, 2002). Previous studies have reported associations between social support and cultural adaptation or cultural identity. For example, Kang (2018) reported an association between social support and cultural conflict in the context of biculturalism, while Ning (2015) found that ethnic alienation among adolescents of the Li ethnic group in Hainan Province was negatively associated with social support.

Research in exercise psychology has also reported associations between physical activity and social support. Physical activity participation has been associated with social-network characteristics and individuals’ experiences of receiving and providing social support (Xu & Li, 2017). More recent evidence further supports this relationship among university students. A meta-analysis of 19 studies found a significant positive association between social support and physical activity among college and university students, while also indicating that the strength of this association may vary according to the source of support and participant characteristics (X. Wang et al., 2024).

Furthermore, existing research has linked social support with cultural identity. Social identification theory suggests that social support is embedded within social relationships and group contexts, as individuals who identify with a social group are more likely to provide and receive support from other group members (Levine et al., 2005). Supportive social relationships may therefore provide a social context in which individuals experience group connection and define themselves in relation to their cultural community. However, the SSRS used in the present study assesses general rather than culturally specific social support. Accordingly, the present study does not assume that social support specifically strengthens local cultural identity; rather, it examines whether general social support is associated with local cultural identity and represents a potential indirect pathway linking physical exercise with local cultural identity. Based on this rationale, the following hypothesis was proposed: H2. There is a significant indirect association between physical exercise and college students’ local cultural identity through social support.

Furthermore, social support encompasses three dimensions—objective support, subjective support, and support utilization—which represent distinct ways in which individuals obtain and engage with social resources (Xu & Li, 2017). Objective support refers to tangible resources or assistance actually available or received through social relationships; such support may reflect greater social embeddedness and provide concrete opportunities for interpersonal connection and group belonging. Subjective support reflects individuals’ perceived acceptance and support from others, which may be associated with a stronger sense of social connection and identity affirmation. Support utilization reflects the active seeking and use of available support and may increase individuals’ engagement with social relationships through which group connection is experienced. Thus, the three dimensions may represent distinct potential pathways linking physical exercise with local cultural identity. Based on this rationale, the following hypothesis was proposed: H3. Physical exercise is indirectly associated with college students’ local cultural identity through the three dimensions of social support—objective support, subjective support, and support utilization.

## 2. Materials and Methods

### 2.1. Participants

This study targeted university students, collecting data via self-administered questionnaires using the Sojump App for non-probability sampling. A non-probability snowball sampling method was employed. An initial group of university students was recruited and invited to complete the questionnaire, and these participants were subsequently asked to share the questionnaire link with eligible classmates and peers, thereby expanding the sample through participant referrals. Throughout the survey, emphasis was placed on the anonymity, voluntariness, and authenticity of responses. Before beginning the survey, participants were informed of the purpose of the study, the anonymous and voluntary nature of participation, and their right to withdraw at any time. Participants were informed that proceeding with the questionnaire would be regarded as providing informed consent.

To establish an appropriate completion-time criterion for data screening, a pilot test was conducted with 29 university students to estimate the time required to complete the questionnaire. The mean completion time was 330.69 s (SD = 101.69 s; median = 312 s), and the shortest observed completion time was 237 s. Based on these results, a completion time of less than 120 s (2 min), which was approximately two standard deviations below the pilot mean and substantially shorter than the shortest completion time observed in the pilot test, was considered indicative of excessively rapid responding. Prior to statistical analysis, all returned questionnaires were screened for response quality. Questionnaires were excluded if they met either of the following criteria: (1) a completion time of less than 2 min; or (2) evidence of patterned responding. Patterned responding was operationally defined as clear mechanical response sequences across the scale items, including selecting the same response option for all items, repeatedly cycling through response categories in a fixed sequence, or selecting the same response category for a fixed number of consecutive items before systematically switching to another category.

Of the 465 questionnaires collected, 53 were excluded, resulting in a final analytic sample of 412 participants. The final sample included 254 male (61.7%) and 158 female (38.3%) participants. The mean age was 19.83 years (SD = 1.39), with participants ranging in age from 17 to 23 years. Regarding academic year, 96 participants (23.3%) were freshmen, 93 (22.6%) were sophomores, 98 (23.8%) were juniors, and 125 (30.3%) were seniors. In terms of place of residence, 233 participants (56.6%) were from urban areas, and 179 (43.4%) were from rural areas. To evaluate the adequacy of the sample size for the hypothesized parallel mediation model, a Monte Carlo power analysis was conducted using Mplus 8.3 (Muthén & Muthén, Los Angeles, CA, USA). In the absence of directly comparable prior estimates, conservative standardized population parameters were specified for the component paths of the indirect associations (X→M = 0.25 and M→Y = 0.25), corresponding to an expected standardized indirect effect of approximately 0.063. The direct association between X and Y was also set to 0.25, and the residual correlations among the three parallel mediators were set to 0.30. Using 10,000 Monte Carlo replications and a significance level of 0.05, a sample size of 200 yielded statistical power of approximately 0.83 for each of the three specific indirect associations, exceeding the conventional 0.80 criterion. Therefore, the final analytic sample of 412 participants was considered adequate for the proposed structural model.

### 2.2. Measurement Tools

#### 2.2.1. Physical Activity Rating Scale

Physical exercise was assessed using the Physical Activity Rating Scale (PARS-3) revised by Liang (1994). Although the official English name of the instrument uses the term “physical activity,” its items assess the intensity, duration, and frequency of participants’ regular physical exercise. Accordingly, in the present study, “physical exercise” is used to refer to the construct assessed by the PARS-3, whereas “exercise volume” refers specifically to the composite score calculated from exercise intensity, duration, and frequency. The PARS-3 does not distinguish among specific exercise types or contexts, such as team versus individual or competitive versus noncompetitive exercise. This 3-item scale measures the intensity, duration, and frequency of physical activity participation. Each item offers 5 response options scored from 1 to 5 points in ascending order of intensity. When evaluating an individual’s physical activity volume, the formula is Activity Volume Score = Exercise Frequency Score × (Exercise Duration Score − 1) × Exercise Intensity Score. Based on this formula, the maximum possible score for physical activity volume is 100 points, and the minimum is 0 points. Specifically, an Activity Volume Score ≤ 19 indicates low activity volume. A score between 20 and 42 indicates moderate activity volume. A score ≥ 43 indicates high activity volume. Previous research has provided evidence for the temporal stability of the PARS-3, with Liang (1994) reporting a test–retest reliability coefficient of 0.82. In the present study, test–retest reliability was additionally examined in the pilot sample. The 29 university students who participated in the pilot survey completed the PARS-3 again two weeks after the initial assessment. The test–retest reliability coefficient for the PARS-3 total score was 0.909, providing preliminary evidence of temporal stability in the context of the present study.

#### 2.2.2. Local Cultural Identity Scale

The “College Student Local Cultural Identity Scale” developed by S. J. Liu (2019) was selected. In the present study, local cultural identity refers specifically to college students’ identification with Chinese culture as operationalized by S. J. Liu’s (2019) College Student Local Cultural Identity Scale. The construct encompasses three theoretically defined dimensions: cultural affect, cultural behavior, and cultural confidence, reflecting individuals’ emotional attachment to, behavioral engagement with, and confidence in Chinese culture. Thus, the term “local cultural identity” is used throughout this article to refer to the construct assessed by this scale and should not be interpreted as identification with a particular province, city, or other subnational local culture. This 12-item scale comprises three dimensions: cultural affect (5 items), cultural behavior (4 items), and cultural confidence (3 items). It employs a 5-point Likert scale ranging from “strongly disagree” to “strongly agree,” scored from 1 to 5 points. Higher scores indicate greater individual local cultural identity. In the present sample, the Cronbach’s α coefficients were 0.944 for cultural affect, 0.918 for cultural behavior, and 0.949 for cultural confidence. For descriptive statistics and bivariate correlation analyses, the total scale score was used as an observed measure of local cultural identity. In the subsequent structural equation modeling, local cultural identity was specified as an overarching latent construct indicated by the three dimension scores of cultural affect, cultural behavior, and cultural confidence, thereby representing the variance shared across the three theoretically defined dimensions.

#### 2.2.3. Social Support Assessment Scale

The Social Support Rating Scale (SSRS), revised by Xiao Shuiyuan and adapted by Xu Wei et al., was selected to measure college students’ social support status (X. D. Wang et al., 1999; Xu & Li, 2017). This scale comprises 10 items covering three distinct dimensions: objective support (3 items), subjective support (4 items), and utilization of support (3 items). The maximum scores for these dimensions are 24, 28, and 12 points, respectively. The total score is calculated by summing the scores of all 10 items, with higher total scores indicating greater levels of social support received. In this study, the Cronbach’s α coefficient for the scale was 0.97. In the present sample, the Cronbach’s α coefficients were 0.801 for objective support, 0.673 for subjective support, and 0.832 for support utilization. Although the internal consistency of the subjective support subscale was slightly below the conventional 0.70 benchmark, coefficient alpha is sensitive to scale length and may be lower for scales containing relatively few items (Cortina, 1993). Given that the subjective support subscale comprised only four items, this coefficient was interpreted with appropriate caution.

### 2.3. Data Processing

Descriptive statistics and Pearson correlation analyses were conducted using SPSS 18.0 (IBM Corp., Armonk, NY, USA). Confirmatory factor analyses (CFAs) and structural equation modeling (SEM) were performed using Mplus 8.3. As supplementary evidence of construct validity in the present sample, item-level CFAs were conducted for the Local Cultural Identity Scale and the Social Support Rating Scale (SSRS). Ordered categorical indicators were estimated using the weighted least squares mean- and variance-adjusted (WLSMV) estimator. All items of the Local Cultural Identity Scale were treated as ordered categorical indicators. For the SSRS, Items 1–4 and 8–10 were treated as ordered categorical indicators, whereas Items 5–7 were retained as quantitative indicators in accordance with the original scoring rules of the instrument.

The subsequent structural model was estimated using maximum likelihood (ML) estimation. Because the structural model was based on scale or dimension scores rather than individual ordinal items, physical exercise, objective support, subjective support, support utilization, and the three dimension scores used as indicators of the latent local cultural identity construct were treated as continuous variables. There were no missing values for the variables included in the analyses; therefore, no imputation or other missing-data procedure was required. Distributional characteristics of the continuous variables included in the structural model were examined using skewness and kurtosis. The absolute skewness values were no greater than 0.564, and the absolute excess kurtosis values were no greater than 1.459, indicating no substantial univariate distributional abnormalities.

STDYX-standardized estimates are reported for the structural paths and indirect associations. Model fit was evaluated using the chi-square statistic (χ^2^), comparative fit index (CFI), Tucker–Lewis index (TLI), root mean square error of approximation (RMSEA), and standardized root mean square residual (SRMR). Given the cross-sectional design, the directional paths in the structural model represent a theoretically proposed ordering for estimating indirect associations rather than evidence of temporal or causal ordering among the variables. Recent methodological work has emphasized that cross-sectional mediation models cannot establish temporal precedence and may be susceptible to temporal bias in estimates of indirect associations (Georgeson et al., 2025). The statistical significance of the indirect associations was evaluated using 95% bias-corrected bootstrap confidence intervals based on 5000 bootstrap samples (Preacher & Hayes, 2008). An indirect association was considered statistically significant when its 95% confidence interval did not include zero. Given the relatively high correlations among some study variables, potential multicollinearity was additionally assessed using variance inflation factors (VIFs) and tolerance values. The Mplus syntax used to estimate the structural model is provided in Supplementary Material S1 to facilitate reproducibility.

To examine the robustness of the main findings to potential demographic confounding, an additional sensitivity analysis was conducted in which sex, age, urban–rural residence, and academic year were included as covariates. Academic year was dummy-coded with first-year students as the reference category. The demographic covariates were specified as predictors of the three dimensions of social support and local cultural identity. The original structural model was retained as the primary analysis, whereas the covariate-adjusted model was used to evaluate the robustness of the findings.

## 3. Results

### 3.1. Measurement Model

As supplementary evidence of construct validity in the present sample, an item-level CFA was conducted for the Local Cultural Identity Scale. The theoretically specified three-factor model comprised cultural affect, cultural behavior, and cultural confidence and was estimated using WLSMV. The model yielded CFI = 0.994, TLI = 0.993, RMSEA = 0.144, 90% CI [0.132, 0.155], and SRMR = 0.042. The STDYX-standardized factor loadings ranged from 0.848 to 0.995 and were all statistically significant (*p*s < 0.001). Although the CFI, TLI, and SRMR indicated good fit, the RMSEA indicated model misfit. The latent correlations among cultural affect, cultural behavior, and cultural confidence were high (r = 0.969–0.975), indicating substantial shared variance across the three theoretically defined dimensions. This pattern is consistent with their use as indicators of an overarching local cultural identity construct in the subsequent structural model. Nevertheless, the high inter-factor correlations also suggest limited empirical distinctiveness among the three dimensions in the present sample and should therefore be interpreted cautiously.

For social support, a three-factor model comprising objective support, subjective support, and support utilization was examined. The WLSMV-estimated model showed a good fit to the data: χ^2^(32) = 71.606, CFI = 0.981, TLI = 0.973, RMSEA = 0.055, 90% CI [0.038, 0.072], and SRMR = 0.031. The STDYX-standardized factor loadings ranged from 0.619 to 0.897 and were all statistically significant (*p*s < 0.001). The latent correlations among the three social support dimensions ranged from 0.503 to 0.699. Together, the good fit of the three-factor model and the moderate-to-strong, but clearly below-unity, latent correlations provided evidence that the three social support dimensions were empirically distinguishable in the present sample. No post hoc modifications were made to the measurement models reported above.

### 3.2. Assessment of Potential Common Method Bias

As a diagnostic assessment of potential common method bias, a single-factor confirmatory factor analysis was conducted by loading all self-reported measurement items onto a single latent factor. The one-factor model showed a poor fit to the data: χ^2^/df = 12.200, CFI = 0.735, TLI = 0.708, RMSEA = 0.165, and SRMR = 0.083. This result suggests that the covariance among the study measures was unlikely to be adequately represented by a single common factor. However, poor fit of the single-factor model does not rule out the presence of common method variance. Therefore, this analysis was treated only as a diagnostic assessment rather than as evidence for the absence of common method bias.

### 3.3. Descriptive Statistics and Correlation Analysis of Physical Exercise, Local Cultural Identity, and Social Support

As shown in Table 1, physical exercise, the three dimensions of social support, total social support, and local cultural identity were significantly correlated with each other. These correlations provide descriptive information regarding the bivariate associations among the study variables. The hypothesized indirect associations were subsequently examined directly using structural equation modeling with bias-corrected bootstrap confidence intervals.

Given the relatively high correlations among some study variables, multicollinearity was further examined. The VIF values for physical exercise, objective support, subjective support, and support utilization were 3.917, 1.576, 3.092, and 1.833, respectively, with corresponding tolerance values of 0.255, 0.635, 0.323, and 0.546. Although physical exercise was strongly correlated with subjective support (r = 0.816), these diagnostics did not indicate severe multicollinearity among the variables included in the structural model.

### 3.4. Structural Model and Indirect Association Analysis

Using physical exercise as the independent variable, objective support, subjective support, and support utilization as three parallel mediators, and local cultural identity as the dependent variable, the hypothesized structural model was estimated. Local cultural identity was modeled as a latent construct indicated by the dimension scores of cultural affect, cultural behavior, and cultural confidence. The model simultaneously estimated the direct association between physical exercise and local cultural identity and the specific indirect associations through the three dimensions of social support. The structural model showed an acceptable fit to the data: χ^2^(11) = 30.038, *p* = 0.002, CFI = 0.993, TLI = 0.986, RMSEA = 0.065, 90% CI [0.038, 0.093], and SRMR = 0.018.

**Figure 1:**
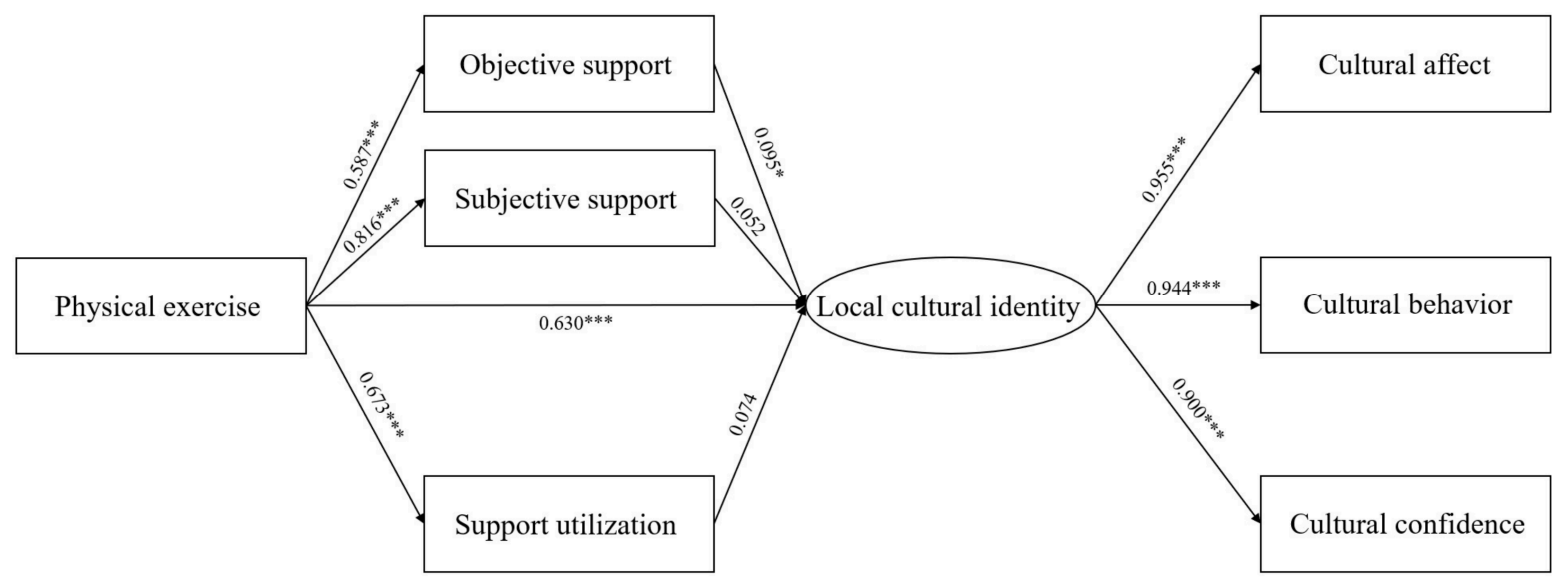
Structural model of physical exercise, social support, and local cultural identity. Note. All coefficients displayed in the figure are STDYX-standardized estimates. Coefficients for paths among physical exercise, the three dimensions of social support, and local cultural identity represent standardized structural path coefficients (β), whereas coefficients from the latent local cultural identity construct to cultural affect, cultural behavior, and cultural confidence represent standardized factor loadings (λ). * *p* < 0.05, *** *p* < 0.001.

As shown in Figure 1, physical exercise was significantly and positively associated with objective support (β = 0.587, *p* < 0.001), subjective support (β = 0.816, *p* < 0.001), support utilization (β = 0.673, *p* < 0.001), and local cultural identity (β = 0.630, *p* < 0.001). Objective support was significantly and positively associated with local cultural identity (β = 0.095, *p* = 0.032), whereas subjective support (β = 0.052, *p* = 0.362) and support utilization (β = 0.074, *p* = 0.103) did not show statistically significant independent associations with local cultural identity.

The significance of the indirect associations was examined using bias-corrected bootstrap confidence intervals based on 5000 bootstrap samples. As shown in Table 2, the total indirect association between physical exercise and local cultural identity through the three dimensions of social support was significant (standardized indirect association = 0.148, SE = 0.056, 95% BC bootstrap CI [0.039, 0.257]). Among the three specific indirect pathways, the indirect association through objective support was significant (standardized indirect association = 0.056, SE = 0.026, 95% BC bootstrap CI [0.005, 0.107]). In contrast, the indirect associations through subjective support (standardized indirect association = 0.042, SE = 0.046, 95% BC bootstrap CI [−0.048, 0.134]) and support utilization (standardized indirect association = 0.050, SE = 0.031, 95% BC bootstrap CI [−0.013, 0.108]) were not statistically significant because their confidence intervals included zero. Thus, the data do not provide sufficient evidence for specific indirect associations through subjective support or support utilization.

Taken together, the significant total indirect association provided support for H2, whereas the pattern of the three specific indirect associations provided only partial support for H3. Specifically, only the indirect association through objective support was statistically significant, while the indirect associations through subjective support and support utilization were not significant. Therefore, the findings do not support the interpretation that all three dimensions of social support individually account for the association between physical exercise and local cultural identity. Overall, H1 and H2 were supported, whereas H3 was partially supported.

### 3.5. Sensitivity Analysis

To examine the robustness of the findings to potential demographic confounding, an additional analysis was conducted adjusting for sex, age, urban–rural residence, and academic year. The covariate-adjusted model showed a good fit to the data: χ^2^(23) = 43.741, *p* = 0.006, CFI = 0.992, TLI = 0.980, RMSEA = 0.047, 90% CI [0.025, 0.068], and SRMR = 0.014. The substantive pattern of results remained unchanged. The indirect association through objective support remained statistically significant (standardized indirect association = 0.057, 95% BC bootstrap CI [0.005, 0.107]), whereas the indirect associations through subjective support (standardized indirect association = 0.036, 95% BC bootstrap CI [−0.055, 0.126]) and support utilization (standardized indirect association = 0.049, 95% BC bootstrap CI [−0.014, 0.107]) remained nonsignificant. The direct association between physical exercise and local cultural identity also remained statistically significant (β = 0.639, *p* < 0.001). These results indicate that the principal findings were robust to adjustment for the measured demographic characteristics.

## 4. Discussion

### 4.1. Association Between Physical Exercise and College Students’ Local Cultural Identity

The findings showed that physical exercise was positively associated with college students’ local cultural identity, and a significant indirect association was also observed through objective support. These findings indicate statistical associations among physical exercise, objective support, and local cultural identity but do not by themselves establish the psychological or social processes underlying these associations. These findings are generally consistent with previous research reporting associations between physical exercise or sport participation and cultural identity (D’Angelo et al., 2019; Doidge et al., 2020; Lee, 2005). In the structural model, local cultural identity was represented as an overarching latent construct indicated by cultural affect, cultural behavior, and cultural confidence. Thus, the observed association with physical exercise should be interpreted as an association with the shared local-cultural-identity construct reflected across these three dimensions, rather than as evidence of distinct associations with any single dimension. Moran’s (2004) experiential learning framework may provide one possible interpretive perspective for understanding the observed association. From this perspective, cultural learning involves engagement with cultural experiences, interpretation of cultural information and concepts, and reflection on the relationship between the self and the cultural community. Applied cautiously to the present context, certain forms of physical exercise, particularly when embedded in social or cultural contexts, may provide experiences associated with cultural awareness, interpretation, and reflection, which could potentially be related to local cultural identity. However, these experiential-learning processes were not directly measured in the present study. Therefore, they should be regarded as theoretically plausible explanations for the observed association between physical exercise and local cultural identity.

### 4.2. Indirect Associations Through Social Support

The structural model showed a significant total indirect association between physical exercise and local cultural identity through the three dimensions of social support. However, among the three specific indirect pathways, only the indirect association through objective support was statistically significant. Although objective support showed a statistically significant positive association with local cultural identity, the magnitude of this association was modest (β = 0.095). Therefore, statistical significance should not be interpreted as indicating a strong association or substantial practical importance. These findings indicate that objective support was the only dimension showing a statistically significant specific indirect association; however, the modest magnitude of its association with local cultural identity suggests that its practical contribution should be interpreted cautiously.

One possible explanation concerns the social contexts that may accompany some forms of physical exercise. Previous research suggests that participation in socially interactive exercise or sport contexts may be associated with opportunities for interpersonal interaction and supportive relationships. Such social experiences could potentially contribute to the observed association between physical exercise and objective support. However, the present study did not assess exercise type, social-network characteristics, interaction frequency, or the specific social contexts in which exercise occurred. Therefore, social-network expansion and increased interpersonal interaction should be regarded as possible explanations for the observed association rather than as processes demonstrated by the present data. This interpretation is consistent with previous research linking supportive relationships with interaction frequency (Wu et al., 2008) and larger social networks with greater opportunities for receiving social support (Wellman & Berkowitz, 1988).

Social Support Resource Theory provides another possible explanation. Social support may contribute to self-definition by embedding individuals within social relationships through which connection, belonging, and identity are experienced (Hobfoll et al., 1990). This theoretical perspective may help explain the observed association between objective support and local cultural identity. However, self-definition was not directly assessed in the present study, and the current findings therefore cannot establish self-definition as the psychological mechanism underlying this association.

A particularly noteworthy finding was that the three dimensions of social support did not show equivalent indirect associations. Among the three specific pathways, only the indirect association through objective support was statistically significant, whereas those through subjective support and support utilization were not significant. This pattern suggests that the role of social support in the association between physical exercise and local cultural identity may depend on the specific form of support rather than reflect social support as a unitary construct. Objective support represents tangible and observable resources or assistance actually available or received through social relationships. One possible interpretation is that socially interactive forms of physical exercise may provide contexts in which individuals have greater opportunities for interpersonal contact and access to concrete forms of support. However, because the PARS-3 assesses exercise volume rather than the social characteristics of exercise participation, the present study cannot determine whether such socially interactive exercise contexts account for the observed indirect association through objective support. Nevertheless, the standardized association between objective support and local cultural identity was modest (β = 0.095), indicating that objective support accounted for only a limited unique association with local cultural identity after physical exercise and the other dimensions of social support were considered. Accordingly, its role should be interpreted cautiously and should not be overstated.

The absence of statistically significant indirect associations through subjective support and support utilization may reflect both substantive and statistical factors. Subjective support reflects perceived support and may depend partly on individuals’ expectations and interpretations of interpersonal relationships, whereas support utilization reflects the behavioral tendency to seek and use available support. Neither necessarily captures whether the support received is directly relevant to cultural identification. In addition, the relatively strong bivariate associations involving subjective support did not translate into a significant unique association with local cultural identity in the parallel mediation model. This discrepancy may partly reflect shared variance among physical exercise and the three dimensions of social support. In a parallel mediation model, each mediator represents its unique association with the outcome after accounting for the predictor and the other mediators. Although physical exercise was strongly correlated with subjective support (r = 0.816), the VIF and tolerance diagnostics did not indicate severe multicollinearity. Thus, shared variance may have reduced the unique contribution of subjective support, but the nonsignificant pathway should not be attributed solely to severe multicollinearity.

Measurement characteristics should also be considered. The internal consistency of the subjective support subscale (Cronbach’s α = 0.673) was lower than that of objective support (α = 0.801) and support utilization (α = 0.832), and the comparatively lower reliability may have attenuated its unique association with local cultural identity. At the same time, the three-factor measurement model for social support showed an acceptable fit, and the latent correlations among the three dimensions (r = 0.503–0.699) indicated that they were empirically distinguishable while sharing meaningful variance. Accordingly, the nonsignificant pathways through subjective support and support utilization should not be interpreted as evidence that these forms of support are unimportant. Future studies using alternative measures and longitudinal designs are needed to determine whether the observed differences reflect substantive distinctions among support types, shared variance among the dimensions, measurement characteristics, or a combination of these factors.

### 4.3. Research Limitations and Future Directions

Several limitations should be considered when interpreting the present findings. First, the cross-sectional design limits conclusions regarding temporal ordering and causality among physical exercise, social support, and local cultural identity. Although significant direct and indirect associations were observed, these findings do not establish causal mediation, and alternative directional relationships or common causes remain possible. Future longitudinal or experimental studies are needed to establish temporal ordering and more rigorously examine these relationships.

Second, the non-probability snowball sampling strategy limits the representativeness and generalizability of the findings. Participants were recruited through peer referrals rather than probability-based sampling, and detailed information on their geographic regions of origin was not collected. The findings should therefore be interpreted as applying primarily to the present sample rather than serving as population-level estimates for Chinese university students. Future studies using probability-based or more geographically diverse samples are warranted.

Third, several measurement limitations should be acknowledged. All principal variables were assessed using self-report measures within the same survey, which may have introduced common method variance and response bias. Although the single-factor CFA did not support a single common factor, it cannot rule out common method variance. In addition, the high latent correlations among cultural affect, cultural behavior, and cultural confidence indicate limited empirical distinctiveness among these dimensions, warranting further examination of the factorial structure of the Local Cultural Identity Scale. The PARS-3 assesses exercise volume through intensity, duration, and frequency but does not distinguish exercise types or social contexts. Accordingly, the present findings cannot determine whether socially interactive and individual forms of exercise differ in their associations with social support and local cultural identity.

Fourth, residual confounding and unmeasured explanatory processes remain possible. Although sensitivity analyses adjusting for sex, age, urban–rural residence, and academic year yielded a similar pattern of findings, other relevant factors were not measured or controlled. Moreover, potential processes discussed in this study, such as experiential cultural learning, social-network expansion, interpersonal interaction, and self-definition, were not directly assessed and should therefore be regarded as theoretically plausible interpretations rather than empirically established mechanisms. Future research should directly assess these processes and incorporate a broader range of relevant covariates.

## 5. Conclusions

The present study found positive associations among physical exercise, objective support, and local cultural identity among Chinese college students. The observed data were also consistent with a significant indirect statistical pathway between physical exercise and local cultural identity through objective support. In contrast, the specific indirect associations through subjective support and support utilization were not statistically significant. These findings suggest that objective support may be relevant to understanding the association between physical exercise and local cultural identity. However, given the cross-sectional design, the observed indirect association should not be interpreted as evidence that physical exercise causally increases objective support or local cultural identity, or that objective support causally mediates this relationship. Longitudinal and experimental research is needed to establish temporal ordering and examine whether the proposed indirect pathway is supported prospectively.

## Figures and Tables

**Table 1:** Descriptive Statistics and Correlations Among the Study Variables (N = 412).

	*M*	*SD*	1	2	3	4	5	6
1. Physical Exercise	37.25	26.62	1.00					
2. Objective Support	8.50	2.74	0.59 ***	1.00				
3. Subjective Support	21.01	3.22	0.82 ***	0.56 ***	1.00			
4. Support Utilization	9.06	2.25	0.67 ***	0.43 ***	0.57 ***	1.00		
5. Total Social Support Score	38.57	6.80	0.85 ***	0.81 ***	0.89 ***	0.77 ***	1.00	
6. Local Cultural Identity	49.23	9.85	0.76 ***	0.51 ***	0.65 ***	0.56 ***	0.70 ***	1.00

Note. *M* = Mean; *SD* = Standard Deviations; *** *p* < 0.001.

**Table 2:** STDYX-Standardized Indirect Associations Between Physical Exercise and Local Cultural Identity (N = 412).

Indirect Association	STDYX-Standardized Estimate	SE	95% BC Bootstrap CI
Total Indirect Association	0.148	0.056	[0.039, 0.257]
Path 1: Physical Exercise → Objective Support → Local Cultural Identity	0.056	0.026	[0.005, 0.107]
Path 2: Physical Exercise → Subjective Support → Local Cultural Identity	0.042	0.046	[−0.048, 0.134]
Path 3: Physical Exercise → Support Utilization → Local Cultural Identity	0.050	0.031	[−0.013, 0.108]

Note. STDYX-standardized estimates are reported. SE = standard error; BC bootstrap CI = bias-corrected bootstrap confidence interval. Confidence intervals were estimated using 5000 bootstrap samples. An indirect association was considered statistically significant when its 95% BC bootstrap confidence interval did not include zero.

## Data Availability

The anonymized data supporting the findings of this study are not publicly available but may be obtained from the corresponding author upon reasonable request, subject to applicable ethical and institutional restrictions. The Mplus syntax used for the primary analyses is provided in the Supplementary Material to facilitate reproducibility. Additional analytical materials may be obtained from the corresponding author upon reasonable request.

## Supplementary Materials

The following supporting information can be downloaded at: https://www.mdpi.com/article/10.3390/bs16091696/s1, S1: Mplus Syntax for the Structural Equation Model.

## References

[B1-behavsci-16-01696] Berry J. W. (1999). Aboriginal cultural identity. Canadian Journal of Native Studies.

[B2-behavsci-16-01696] Chen L. (2021). Cultural identity is the deepest form of recognition. People’s Daily.

[B3-behavsci-16-01696] Commission of the European Communities (2007). White paper on sport [Report].

[B4-behavsci-16-01696] Cortina J. M. (1993). What is coefficient alpha? An examination of theory and applications. Journal of Applied Psychology.

[B5-behavsci-16-01696] D’Angelo C., Corvino C., De Leo A., Martín R. S. (2019). How can performing arts and urban sport enhance social inclusion and interculturalism in the public space? An explorative ethnography of migrants’ performances in the city of Milan, Italy. Comunicazioni Sociali.

[B6-behavsci-16-01696] Doidge M., Keech M., Sandri E. (2020). Active integration’: Sport clubs taking an active role in the integration of refugees. International Journal of Sport Policy and Politics.

[B7-behavsci-16-01696] Donnelly P., Donnelly J. (2002). The role of recreation in promoting social inclusion.

[B8-behavsci-16-01696] Elbe A.-M., Hatzigeorgiadis A., Morela E., Ries F., Kouli O., Sanchez X. (2018). Acculturation through sport: Different contexts different meanings. International Journal of Sport and Exercise Psychology.

[B9-behavsci-16-01696] Georgeson A. R., Alvarez-Bartolo D., MacKinnon D. P. (2025). A sensitivity analysis for temporal bias in cross-sectional mediation. Psychological Methods.

[B10-behavsci-16-01696] Guerin P. B., Diiriye R. O., Corrigan C., Guerin B. (2003). Physical activity programs for refugee Somali women: Working out in a new country. Women & Health.

[B11-behavsci-16-01696] Han Z. (2010). On national identity, ethnic identity, and cultural identity: An analysis and reflection based on historical philosophy. Journal of Beijing Normal University (Social Sciences Edition).

[B12-behavsci-16-01696] Hobfoll S. E., Freedy J., Lane C., Geller P. (1990). Conservation of social resources: Social support resource theory. Journal of Social and Personal Relationships.

[B13-behavsci-16-01696] Ito E., Nogawa H., Kitamura K., Walker G. J. (2011). The role of leisure in the assimilation of Brazilian immigrants into Japanese society: Acculturation and structural assimilation through judo participation. International Journal of Sport and Health Science.

[B14-behavsci-16-01696] Ji S., Chen S., Yang X., Liu T., Wang X. (2024). The effect of sports participation on the social identity of Chinese university students. Frontiers in Psychology.

[B15-behavsci-16-01696] Kang T. H. (2018). Who am I? Migrant workers’ bicultural identity integration, social support, and social maladjustment. Social Behavior and Personality: An International Journal.

[B16-behavsci-16-01696] Lee Y. (2005). A new voice: Korean American women in sports. International Review for the Sociology of Sport.

[B17-behavsci-16-01696] Levine R. M., Prosser A., Evans D., Reicher S. D. (2005). Identity and emergency intervention: How social group membership and inclusiveness of group boundaries shape helping behavior. Personality and Social Psychology Bulletin.

[B18-behavsci-16-01696] Liang D. Q. (1994). Stress levels among college students and their relationship with physical exercise. Chinese Journal of Mental Health.

[B19-behavsci-16-01696] Liu J. (2021). Pathways for cultivating Chinese cultural identity among youth in Guangdong, Hong Kong, and Macao through Lingnan sports culture. Guangxi Social Sciences.

[B20-behavsci-16-01696] Liu S. J. (2019). A study on the relationship between international perspectives and local cultural identity among college students. Master’s thesis.

[B21-behavsci-16-01696] Malecki C. K., Demaray M. K. (2002). Measuring perceived social support: Development of the Child and Adolescent Social Support Scale. Psychology in the Schools.

[B22-behavsci-16-01696] Moran P. R. (2004). Teaching culture: A practical approach.

[B23-behavsci-16-01696] Ning S. W., Chinese Psychological Society (2015). The influence of ethnic identity on cultural alienation among Li ethnic youth: The mediating role of family intimacy and social support. Proceedings of the 18th national psychology conference.

[B24-behavsci-16-01696] Padilla A. M., Perez W. (2003). Acculturation, social identity, and social cognition: A new perspective. Hispanic Journal of Behavioral Sciences.

[B25-behavsci-16-01696] Patricia A. B. (2010). Black cultural advancement: Racial identity and participation in the arts among the black middle class. Ethnic and Racial Studies.

[B26-behavsci-16-01696] Preacher K. J., Hayes A. F. (2008). Asymptotic and resampling strategies for assessing and comparing indirect effects in multiple mediator models. Behavior Research Methods.

[B27-behavsci-16-01696] Rosenberg D., Feigin N., Talmor R. (2003). Perceptions of immigrant students on the absorption process in an Israeli physical education and sport college. European Journal of Physical Education.

[B28-behavsci-16-01696] Rudd A. (2005). Which character should sport develop?. Physical Educator.

[B29-behavsci-16-01696] Schwartz S. J., Montgomery M. J., Briones E. (2006). The role of identity in acculturation among immigrant people: Theoretical propositions, empirical questions, and applied recommendations. Human Development.

[B30-behavsci-16-01696] Sun C., Zhang G. L. (2020). The influence of physical activity on adolescents’ interpersonal skills: A chain mediation effect of body self-esteem and global self-esteem. Chinese Journal of Sports Medicine.

[B31-behavsci-16-01696] Sun C. C., Deng X. H., Song Z. P. (2018). Globalization and nationalization: Cultural identity of traditional ethnic sports in China. Journal of Physical Education.

[B33-behavsci-16-01696] Wang X., Yang X., Juzaily bin Mohd Nasiruddin N., Wei S., Dong D., bin Samsudin S. (2024). Social support and physical activity in college and university students: A meta-analysis. Health Education & Behavior.

[B32-behavsci-16-01696] Wang X. D., Wang X. L., Ma H. (1999). Manual of mental health assessment scales (revised edition).

[B34-behavsci-16-01696] Wellman B., Berkowitz S. D. (1988). Social structures: A network approach.

[B35-behavsci-16-01696] Wu Y. H., Li Z. F., Zeng W. (2008). A study on the correlation between social support and physical exercise among college students in China. Journal of Shandong Institute of Physical Education.

[B36-behavsci-16-01696] Xu W., Li Y. (2017). The effect of physical exercise on depression among female college students: The multiple mediating role of three dimensions of social support. Chinese Journal of Sports Medicine.

[B37-behavsci-16-01696] Zheng X., Wang L. (2005). Cultural identity, social orientation, and subjective well-being among Chinese international students. Psychological Development and Education.

[B38-behavsci-16-01696] Zhou X. H. (2008). Identity theory: Analytical approaches from sociology and psychology. Social Sciences.

