# Supplementary material for: The Association Between Physical Exercise and Local Cultural Identity: Indirect Associations Through Three Dimensions of Social Support
Source: Behav Sci (Basel). 2026 Sep 20;16(9):1696. doi: 10.3390/bs16091696 (PMC13603725; doi:10.3390/bs16091696)
Supplement: Supplementary file 1 [file behavsci-16-01696-s001.zip › behavsci-4483842-supplementary.pdf]

Supplementary Material S1. Mplus Syntax for the Structural Equation Model

The following Mplus syntax was used to estimate the parallel indirect-association model reported in the manuscript. Variable labels correspond to physical exercise (X), objective support (KG), subjective support (ZG), support utilization (LYD), and the three indicators of latent local cultural identity (YZ1–YZ3).

```
TITLE: Parallel indirect-association model;

DATA:

  FILE IS data.dat;

VARIABLE:

  NAMES ARE X KG ZG LYD YZ1 YZ2 YZ3;

ANALYSIS:

  ESTIMATOR = ML;

  BOOTSTRAP = 5000;

MODEL:

  RT BY YZ1 YZ2 YZ3;

  KG ON X (a1);

  ZG ON X (a2);

  LYD ON X (a3);

  RT ON X (c)

     KG (b1)

     ZG (b2)

     LYD (b3);

MODEL INDIRECT:

  RT IND KG X;

  RT IND ZG X;

  RT IND LYD X;

OUTPUT:

  CINTERVAL(BCBOOTSTRAP);

  STANDARDIZED;
```
